# Lipoprotein(a) in patients with breast cancer after chemotherapy: exploring potential strategies for cardioprotection

**DOI:** 10.1186/s12944-023-01926-9

**Published:** 2023-09-22

**Authors:** Ziqing Wang, Jian Li

**Affiliations:** Department of Cardiology, The Affiliated Hospital of Qingdao University, No.1677 Wutai Mountain Road, Qingdao, 266000 China

**Keywords:** Cardiovascular disease, Lipoprotein(a), Measurements, PCSK9 inhibitors

## Abstract

Developments in neoadjuvant and adjuvant chemotherapy (CHT) have led to an increase in the number of breast cancer survivors. The determination of an appropriate follow-up for these patients is of increasing importance. Deaths due to cardiovascular disease (CVD) are an important part of mortality in patients with breast cancer.

This review suggests that chemotherapeutic agents may influence lipoprotein(a) (Lp(a)) concentrations in breast cancer survivors after CHT based on many convincing evidence from epidemiologic and observational researches. Usually, the higher the Lp(a) concentration, the higher the median risk of developing CVD. However, more clinical trial results are needed in the future to provide clear evidence of a possible causal relationship. This review also discuss the existing and emerging therapies for lowering Lp(a) concentrations in the clinical setting. Hormone replacement therapy, statins, proprotein convertase subtilisin/kexin-type 9 (PCSK9) inhibitors, Antisense oligonucleotides, small interfering RNA, etc. may reduce circulating Lp(a) or decrease the incidence of CVD.

## Introduction

**Fig. 1:**
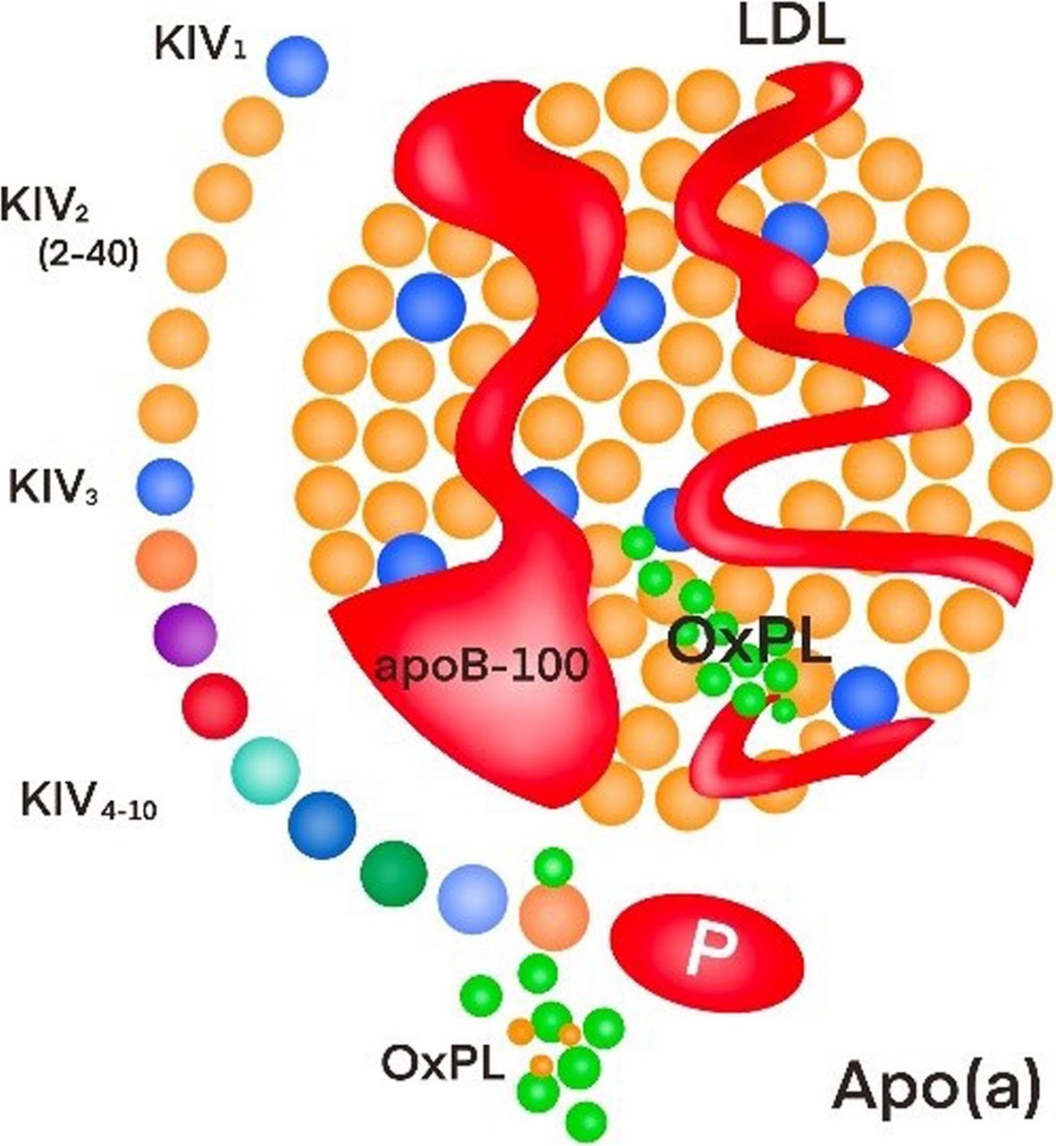
The structure of Lp(a)

Lp(a) is a composite microparticle that exists in body serum; it consists of apolipoprotein B-100 (apoB-100) molecule from the low-density lipoprotein (LDL) class lipoproteins that combines with the macromolecular weight glycoprotein (apolipoprotein a(apo(a)) [1, 2]. Apo(a) is hallmark protein constituent of Lp(a) that has a disulfide protein linkage to apoB-100. There is strong expression of the Lp(a) gene in the liver [3]. Lp(a) is the most genetically regulated lipoprotein, as more than 90% of the concentration is determined by genes [2]. The circulating Lp(a) concentration is significantly affected by the LPA locus coding for apo(a) and lack-of-function genetic mutations in the APOE locus and PCSK9 R46L [4, 5]. Lp(a)/apo(a) is endocytosed by lattice-protein-mediated endocytosis and subsequently enters the lysosome to be degraded [6] Fig. 1.

Lp(a) is formed by a high molecular weight glycoprotein (apo(a)) that combines with the apoB-100 molecule of the LDL class of lipoproteins.

Moreover, Lp(a) is an individually predictive factor of CVD. Globally, 20–30% of the population exhibits Lp(a) levels of over 30 mg/dl [7, 8]. High levels of Lp(a) are also remarkably related to the risk of coronary heart disease [9]. Lp(a) ≥ 125 nmol/L indicates an elevated risk for atherosclerotic cardiovascular disease (ASCVD), especially at higher Lp(a) levels [10]. The average and median levels of Lp(a) vary across racial and ethnic populations, and Lp(a) concentrations are 12% more in females [4, 11]. Although there were notable variations in the average concentrations of Lp(a) between races, the estimated hazard ratios were similar for each equal increase in Lp(a) levels in White, Black as well as South Asian populations. The risk gradient of Lp(a) distribution was similar to that of high-density lipoprotein (HDL) or LDL cholesterol concentrations. Among people without ASCVD, the risk of ASCVD events within 10 years is greater in people with Lp(a) concentrations above 150 nmol/L than in other groups [12].

LDL-C is the primary driver of atherosclerotic CVD [13]. All LDL particles play an atherogenic role to some extent. Evidence suggesting causal roles of additional apoB-containing lipoproteins in ASCVD is increasing [14]. HDL/apoAI may perform an anti-inflammatory and antioxidant function in atherosclerotic plaques by promoting cholesterol efflux, attenuating intraplaque oxidative modifications of LDL, and inhibiting LDL-driven inflammatory processes to slow plaque progression [15].

According to GLOBOCAN 2020 estimates of cancer morbidity and mortality, breast cancer has become the most prevalent cancer for women. The global cancer burden is projected to continue to increase through 2040 [16]. Therefore, it is essential to prevent cancer and improve its prognosis. Neoadjuvant CHT has become a common therapy in early-stage breast cancer [17]. Due to advances and developments in cancer treatment, increasing numbers of patients with cancer are given hope of long-term survival. However, many researches have indicated that current breast cancer treatment may adversely affect the health of the cardiovascular system for the remaining survival time of breast cancer survivors and may contribute to disorders of lipid metabolism [18, 19]. This review summarizes studies on changes in Lp(a) level after CHT among patients with breast cancer and assesses their risk of CVD. Furthermore, we discuss the current and emerging treatments for decreasing Lp(a) concentrations in the clinical setting. The purpose of this review is to address the practical implications of the results and explore potential strategies for cardioprotection for cancer survivors.

## Lipoprotein(a) in breast cancer after chemotherapy

Early adjuvant CHT for breast cancer is commonly used with anthracyclines and paclitaxel, which can improve both disease-free and general survival rates of patients [20, 21]. Many of adjuvant therapies used for breast cancer have variable negative impacts on the cardiovascular system [22]. It has been shown that anthracyclines used for breast cancer can lead to bone marrow suppression and disorders of lipid metabolism during the early stages of treatment. In addition, in some studies, a higher risk of cardiotoxicity, a reduction in cardiac function, and an elevated risk of cardiac failure and cardiomyopathy can occur during the later stages of CHT [23, 24]. For breast cancer survivors, radiation therapy can also lead to an elevated risk of heart failure, CAD, and cardiovascular death [25].

A prospective study conducted by Jordana Carolina Marques Godinho-Mota et al. included 99 women with recently diagnosed with breast cancer. Their outcomes showed that CHT (anthracyclines with taxanes) was related to a rise in lipid-related markers but a decline in high-density lipoprotein cholesterol (HDL-C) levels [26]. Recently, many investigators have been interested in the influence of (neo) adjuvant CHT on levels of circulating lipid, such as dyslipidemia. Moreover, only a small number of people are concerned about the impacts of neoadjuvant CHT on Lp(a) indicators. Lp(a) is an independently significant contributor to risk that predicts the severity of emerging CVD among postmenopausal women. This suggests that Lp(a) is probably the key to the preventive and therapeutic lipid aspects of such patients [27].

Dating back as far as 1996, T Saarto et al. performed adjuvant CHT in 59 patients with confirmed breast cancer and measured their Lp(a) concentrations before and after treatment. The findings demonstrated that circulating Lp(a) levels were elevated substantially only for patients who developed permanent amenorrhea [28]. It is hypothesized that this may be due to the negative effect of ovarian decline induced by chemotherapeutic agents on Lp(a). Patients with declining ovarian function exhibit more risk factors for CVD (hypertension, obesity, etc.) [29]. Lu [30] conducted a similar study and observed that patients treated with an anthracycline-based CHT had considerably increased Lp(a) concentrations before the last cycle. Patients treated with an anthracycline-plus-paclitaxel CHT had decreased Lp(a) levels following the first cycle, but these levels elevated before the final cycle of CHT. Prolonged CHT may also result in variations in LDL-C and Lp(a) concentrations. However, no statistically considerable difference in Lp(a) levels were observed in the studies of Sharma M [20] and Qu [31].

Some studies have shown that CHT markedly changes circulating lipid concentrations in breast cancer survivors, but the roles of different chemotherapeutic agents on lipids or lipoproteins are different. The majority of relevant studies had the same results [20, 32, 33]. ApoA1 and HDL-C concentrations were greatly lower and LDL-C, Triglycerides (TG), serum total cholesterol and apoB (a component of Lp(a)) concentrations were considerably higher [20, 26, 34–36]. Giskeødegård GF's study revealed a marked increase in TG and very LDL-related cholesterol and lipids concentrations, compared to other lipids [37]. Patients with premenopausal breast cancer seem to be more vulnerable to this change [38]. This may be because younger patients have higher sex hormone levels and superior lipid metabolism, so plasma lipid concentrations may be more sensitive to chemotherapeutic drugs [35] Table 1.

**Table 1:** Summary of studies on the measurement of Lp(a) in patients with breast cancer after chemotherapy

Year	Author	Population type	Treatment	Conclusion	Ref
1996	Saarto, T	59 premenopausal women	Cyclophosphamide, methotrexate, and 5-fluorouracil	Lp(a) was significantly higher	[28]
2016	Sharma M	12 women (aged between 25 and 65)	Doxorubicin, cyclophosphamide, epirubicin,5’-fluorouracil and docetaxel	No significant difference in Lp(a)(*P* > 0.05)	[20]
2020	Qi Lu	1016 premenopausal and 627postmenopausal women and 93 peri-menopausal women	Anthracycline-based and taxane-based	Lp(a) was significantly higher(*P* < 0.05)	[30]
2020	Fanli Qu	216 premenopausal and 317 postmenopausal women	Docetaxel, epirubicin and cyclophosphamide	No distinct difference in Lp(a)(*P* > 0.05)	[31]

### Cardiovascular risk among breast cancer survivors

When cancer survivors have longer life expectancy and are treated more cumulatively with cytotoxic treatments, they are more in danger of dying from CVD [39]. Jennifer L Patnaik's work counted the percentage distribution of the main reasons for death among patients with breast cancer aged 66 and above. They discovered that in the study population, CVD was a major reason for death (15.9%), and the second reason was breast cancer (15.1%) [40]. Abdel-Qadir H also found that CVD (16.9%) surpassed breast cancer (14.6%) as the major cause of death for the same population at 10 years after diagnosis [41]. Breast cancer survivors may have a greater risk on atherosclerosis, as this risk was 2.4 times higher than that for women without breast cancer. Additionally, patients older than 45 years had a higher prevalence of metabolic syndrome (54.2% vs. 37.0%) and diabetes (19.8% vs. 6.8%) [42, 43]. CVD is usually a delayed effect of therapy. The disease usually occurs about seven years after treatment for breast cancer has been completed [44].

Roberta Florido examined the incidence of CVD for cancer survivors in the ARIC study. The study involved 12,414 participants and the prevalence of breast cancer was greatest (35%) for women in this group. Independent of traditional cardiovascular risk factors, CVD risk was markedly higher in cancer survivors than in people without cancer (37%); for example, breast cancer was significantly associated with CVD risk [45].

### Effects of Lp(a) on the cardiovascular system

There has been a great amount of work devoted to the impacts of Lp(a) on the cardiovascular system. Convincing evidence from epidemiologic and observational studies indicate higher Lp(a) concentrations have a possible causal relationship with calcified aortic valve stenosis, peripheral arterial disease and ischemic stroke [46–48]. In addition, a Phenome-wide Mendelian randomization study demonstrated that a greater concentration of circulating Lp(a) was statistically relevant to an enhanced risk of some circulatory diseases, including various heart diseases, hypertension, and cerebrovascular diseases, and certain endocrine diseases, including hypercholesterolemia, hyperlipidemia and type 2 diabetes [49].

As mentioned above, Lp(a) is made up of an apo(a) and an LDL-like fraction, which establishes a nuclear connection between the thrombosis process (mediated by the apo(a) fraction) and the atherosclerosis process (mediated by the LDL-like fraction) [50]. It is generally acknowledged that elevated Lp(a) levels are independently linearly correlated with calcific aortic stenosis and CVD [47, 51]. Normally, the greater the Lp(a) concentration is, the higher the median hazard of developing CVD [47]. This is probably contributed by the pro-atherosclerosis and pro-inflammatory roles of Lp(a). Some inflammatory diseases, including rheumatoid arthritis and systemic lupus erythematosus, are related to increased Lp(a) concentrations [52, 53].

There are large clinical studies suggesting that raised Lp(a) concentrations may lead to the incidence of venous thromboembolism, which is attenuated by PCSK9 inhibition [54]. In contrast, Michael B. Boffa et al. indicated that Lp(a) was not a risk contributor to the formation of venous thrombosis but promotes the platelet activation and aggregation as well as the progression of rupture-prone plaques, leading to atherosclerotic events [50, 55]. However, more experiments are needed to demonstrate thrombogenic function of Lp(a) because the pathological mechanisms behind it remain elusive.

## Therapeutic reduction of lipoprotein(a)

Despite the importance of increased Lp(a) concentrations in ASCVD, no lipid-lowering agent has thus far been shown to reduce CVD risk by significantly lowering Lp(a) concentrations [56]. Particularly, PCSK9 monoclonal antibodies are presently approved drugs for use that have been suggested to reduce cardiovascular event risk and Lp(a) concentration [54]. In the 25,096 participants of FOURIER trial, O'Donoghue ML measured Lp(a) concentrations and found that evolocumab (a monoclonal immunoglobulin that binds specifically to human PCSK9) markedly decreased Lp(a) concentrations by an average of 26.9%. Among patients with Lp(a) concentrations greater than median, evolocumab decreased mortality risk from myocardial infarction and coronary heart disease by 23% [57]. Moreover, in the prolonged tracking study, patients randomized to receive evolocumab had a 20% lower incidence of mortality from cardiovascular causes, myocardial infarction or ischemic stroke than the placebo [58]. Functions of PCSK9 inhibitors have been similarly substantiated in other studies [59–62] Table 2.

**Table 2:** Impact of different therapeutic agents on Lp(a) levels and therapeutic roles for breast cancer

Therapeutic agent	Population type	Change in Lp(a)	Therapeutic roles for breast cancer
Hormone replacement therapy	Post-menopausal women	-25% [63]	Not applicable
Statins	Randomized population	8.5–19.6% [64]	Delays the progression and prevents its recurrence
Aspirin	Randomized population	Inconclusive results but can reduce the CVD risk [65]	Reduces the incidence and the chance of cancer metastasis
PCSK9 inhibitors	Patients with high baseline Lp(a)	-24.5–29.5% (more significant in those with baseline Lp(a) of ≤ 125 nmol/l) [66]	Not applicable
Lipoprotein apheresis	Patients with high baseline Lp(a)	-60–70% [67]	Not applicable
Mipomersen	Patients with high baseline Lp(a)	-26.4% [68]	Not applicable
AKCEA-APO(a)-LRX	Patients with baseline Lp(a) > 60 mg per deciliter	-50–80% [69]	Not applicable
IONIS-APO(a)	Patients with baseline Lp(a) ≥ 75 nmol/L	-60–80% [70]	Not applicable
SLN360	Patients with baseline Lp(a) ≥ 150 nmol/L	Dose dependent ( -10–98%) [71]	Not applicable
Olpasiran(AMG890)	Patients with high baseline Lp(a)	-70–97% [72]	Not applicable
Inclisiran	Patients with high baseline Lp(a)	Inconclusive results	Not applicable

### Effect of HRT on Lp(a)

At one time, hormone replacement therapy (HRT) was a common part of drug therapies used for postmenopausal women. The use of HRT declined dramatically with the Women's Health Initiative in 2002 [73]. Several studies demonstrate that HRT significantly reduces Lp(a) concentrations in postmenopausal women [74]. In the analysis of the HERS study, the reducing impact of progestin and estrogen on Lp(a) among postmenopausal women was greatest in the 4th quartile of Lp(a) elevation (55 to 236 mg/dL) [75]. S R Salpeter summarized and compared the influence of HRT on Lp(a) in randomized controlled trials from April 1966 to October 2004, with an average 25% reduction in Lp(a) [-25.0% [CI, -32.9 to -17.1%)] [63]. However, HRT may prevent CAD in younger (age < 60 years) menopausal women, but in older menopausal women (age > 60 years), HRT may increase their risk of CVD during the first 1–2 years of use [76]. Because HRT will increase C-reactive protein and prothrombin concentrations, it is associated with a systemic inflammatory response. Although it reduces Lp(a) concentrations, it cannot reduce the incidence of getting coronary heart disease [77].

### Statins

Statins has significantly improved cardiovascular outcomes and is currently an essential approach for lipid-lowering therapies [78]. Statins improve endothelial nitric oxide synthase activity. It also can increase atherosclerotic plaque stability by inhibiting the production of isoprenoid intermediates in the cholesterol biosynthesis pathway [79]. Thus, it plays its cardiovascular protective role, which is referred to as “pleiotropic effects”. Moreover, statins have been suggested to inhibit the growing and surviving of tumor cells, delaying the progression of breast cancer and preventing its recurrence [80]. Breast cancer survivors also have improved survival rates by using statins [81]. The clinical routine use of statins to prevent cardiotoxicity caused by anthracyclines is currently under discussion.

In an analysis of six randomized studies, statins markedly increased circulating Lp(a) levels. The average percentage changes at baseline ranged from 18.7% to 24.2% in the group using atorvastatin and 11.6% to 20.4% in the group using pravastatin [64, 82]. The researchers found that numerous patients using statins for prevention of CVD still suffered cardiovascular events despite achieving target levels of LDL-C [83]. Plasma Lp(a) levels become a powerful indicator of remnant CVD risk when CVD risk is decreased due to elevated LDL-C concentrations [7]. Despite treatment with statins, Lp(a) has an independent deleterious effect on patients [84]. Angela Pirillo raises the question of whether statins causing elevated Lp(a) levels are of clinical value. It can be argued that we should consider the absolute level of Lp(a) and that percentages can be misleading. Studies indicate that a substantial decrease in absolute Lp(a) concentrations is needed to obtain the risk of CVD reduction comparable to that of a decline in LDL-C concentrations of 1 mmol/L [85]. In a study by de Boer et al., statin therapy did not change the CVD risk related to Lp(a) compared with that of the placebo [86]. We consider that statins are still recommended for patients with higher Lp(a) concentrations because it decrease the incidence of CVD. Furthermore, we need more studies in the future to investigate the function of hypolipidemic drugs in the metabolism of Lp(a).

### Aspirin

Aspirin decreases apo(a) gene transcription, resulting in decreased Lp(a) production by hepatocytes [87]. In the primary prevention of CVD events, treatment by aspirin has been demonstrated to decrease Lp(a)-mediated atherosclerotic thrombotic events [65, 88]. The reduction in ASCVD risk by aspirin may be due to a reduction in Lp(a) levels. No evidence exists to definitively confirm that this treatment can decrease Lp(a) concentrations. Aspirin is used as a primary prevention for lowering the incidence of CVD in people with higher Lp(a) levels [65]. However, it was also discovered to lower the incidence of breast cancer, so its use is becoming a rising trend in cancer control [89, 90]. Aspirin decreases the chance of metastatic cancer and improves the survival rate of patients with breast cancer [91]. In postchemotherapy survivors, treatment with aspirin could achieve benefits in terms of prognosis and prolonged survival, but multicenter clinical trials are needed to verify this assumption.

### PCSK9 inhibitors

PCSK9 is considered to be an appealing target for treating dyslipidemia [92]. PCSK9 contributes to the degradation of hepatic low-density lipoprotein receptor (LDLR). It also has a function to metabolize circulating Lp(a) [93]. PCSK9 mediates the degradation of low density lipoprotein receptor-related protein 1 in HEK293 and HepG2 cells and mouse B16 melanoma cells. Independent of LDLR, PCSK9 promotes low density lipoprotein receptor-related protein 1 degradation [94].

PCSK9 inhibitors, which include evolocumab and alirocumab, can significantly improve the lipid profile of people at high cardiovascular risk [62, 95]. Evolocumab has be proven to decrease LDL-C concentrations to 30 mg per deciliter and reduce the incidence of CVD by inhibiting PCSK9 [96]. PCSK9 mAbs are effective in safely lowering Lp(a) concentration clinical practice, especially in patients with increased Lp(a) concentrations [97, 98]. Accordingly, monoclonal antibodies that suppress PCSK9 have become a hopeful choice for reducing Lp(a) levels [99]. Moreover, the net meta-analysis further indicated that therapy with alirocumab decreased total cause mortality rate and serious adverse events (SAEs), and evolocumab treatment decreased the risk of myocardial infarction [100].

Nevertheless, the mechanism whereby PCSK9 inhibitors decrease elevated Lp(a) concentrations remains controversial.

First, Alirocumab (PCSK9 inhibitor) accelerates the catabolism of Lp(a). The mechanism is likely to a significant upregulation of LDLR and decreased the competition for these receptors for Lp(a) and LDL particles [101]. PCSK9 associates with the epidermal growth factor structural domain A of LDLR, guiding its destruction into endosomes or lysosomes [92]. Rocco Romagnuolo proposed that LDLR is a PCSK9-regulated Lp(a) clearance receptor. They found that PCSK9 inhibition results in a combination of a significantly lower LDLR abundance and LDL in the supraphysiologic liver, thus revealing that LDLR is an important pathway for Lp(a) clearance [6].

If the LDLR pathway completely explains Lp(a) clearance, it can be expected that PCSK9 inhibitor treatment will uniformly decrease Lp(a) and LDL-C in the same individual in 2:1 ratio (consistent reduction) [102]. In a clinical trial, there was no clear correlation between decrease in Lp(a) and LDL-C after treatment with PCSK9 inhibitors [97]. This inconsistent decline may indicate that PCSK9 inhibitor treatment decreases plasma Lp(a) by an alternative route of LDL receptor removal. This presumption was further confirmed by the widespread incidence of inconsistent Lp(a) and LDL-C responses following treatment with alirocumab that was identified in a postevent summary analysis from the ODYSSEY phase 3 clinical trial [102].

Shapiro MD also proposed that Lp(a) removal is not only modulated through the LDLR pathway, but that there may be other mechanisms at play. It may also be regulated by the apo(a) isomer size. The length of the kringle type 4 chain compared to other receptors is the primary determining factor of its capacity to bind LDLR. Their conclusions indicate that patients with bigger apo(a) subtypes may have a stronger Lp(a) reduction response after treatment with evolocumab [103]. The extent to which PCSK9 inhibitors decrease Lp(a) concentrations varies widely. To investigate this variability, Valentin Blanchard evaluated the correlation between Lp(a) concentration and apo(a) size after PCSK9i treatment in 268 patients. The size of apo(a) was found to act as an independent deciding factor of the response to PCSK9i, and Lp(a) levels decreased by 3% for each additional kringle structural domain [104].

**Fig. 2:**
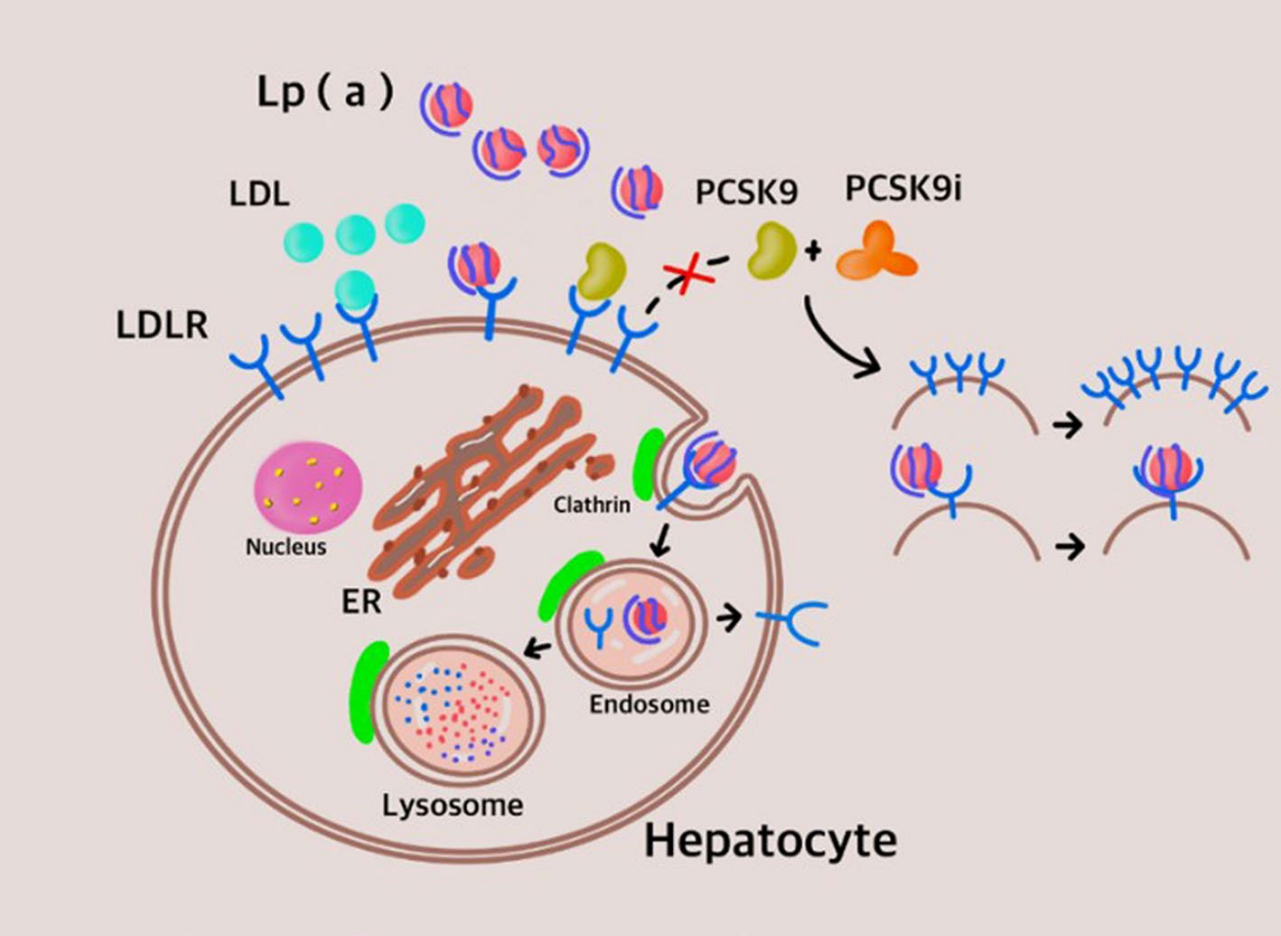
A model of PCSK9 inhibitor receptor-mediated Lp(a) catabolism

Additionally, in Croyal M’s study that investigated both lack of function and gain of function Lp(a) dynamics in PCSK9 mutant patients, the main finding was that the rate of absolute VLDL-apoE production was positively related to the rate of absolute Lp(a)-apo(a) production. They hypothesized that variations in apoE levels in Lp(a) precursors work on the binding of apo(a)-apoB100, while at the same time it is possible that the impact of PCSK9 on Lp(a) correlate with its effect of apoE metabolism [5, 105] Fig. 2.

The mechanism for lowering Lp(a) concentrations by inhibiting PCSK9 has not been clarified. One of the most supported hypotheses is that LDLR is a PCSK9-regulated Lp(a) clearance receptor. Lp(a) can combines with LDLR and compete against LDL. Internalization of Lp(a) depends on lattice-protein-coated pits in the cell, and its degradation occurs in the lysosome. Lp(a) is not bound to PCSK9. PCSK9 inhibition resulted in increased LDLR abundance and the upregulation of LDLR activity in hepatocytes, specifically increasing ability of the Lp(a) molecule to bind with affinity to LDLR and enhancing internalization of LDLR/Lp(a) complexes in hepatocytes.

### Lipoprotein(a) apheresis

Lipoprotein apheresis can affect multiple lipoproteins. Lipoprotein apheresis provides effective reduction of Lp(a) and LDL apoB-100 concentrations by about 60 to 70% [106, 107]. Lp(a) apheresis is a method of immunoadsorption specific for Lp(a) that will only decrease Lp(a) concentrations [108, 109]. A prospective controlled clinical trial using Lp(a) apheresis showed progressive regression of coronary atherosclerosis after weekly elimination of Lp(a) over an 18-month period [107]. Lipoprotein apheresis is a useful therapy for reducing Lp(a) concentrations and is well-tolerated. However, it is expensive and lacks randomized controlled trials, making its widespread use difficult.

### Antisense oligonucleotides

#### Mipomersen

Mipomersen is a type of second-generation antisense oligonucleotide; it is a twenty-polymer oligonucleotide that is compatible with the human-specific apoB-100 messenger RNA coding region. Mipomersen suppresses the synthesis of apoB-100, thereby reducing Lp(a) concentrations in patients at higher CVD risk [110]. In Raal FJ’s research, Lp(a) concentrations were significantly lower in a group of patients with homozygous familial hypercholesterolemia who were previously treated with lipid-lowering medications, including high-dose statins, by taking 200 mg mipomersen weekly [111].

#### AKCEA-APO(a)-LRX

AKCEA-APO(a)-LRX treatment was given to 286 patients with higher Lp(a) levels and preexisting CVD for 6 to 12 months, and the outcomes of the study suggested AKCEA-APO(a)-LRX led to a dose-dependent reduction in Lp(a) concentrations [69].

#### IONIS-APO(a)

In two randomized and double-blind trials conducted by Viney NJ, IONIS-APO(a) was indicated that decreased Lp(a) concentrations in volunteers by 60–80% and was positively correlated with concentration [70].

### siRNA

#### SLN360

SLN360 is a small interfering RNA (siRNA) targeting LPA messenger RNA. A dose-dependent decrease in Lp(a) levels was noted after therapy on SLN360 in the phase 1 study in 2022 [71].

#### Olpasiran(AMG890)

Olpasiran is a synthetic siRNA aimed at specifically inhibiting the translating of LPA messenger RNA in hepatic cells that effectively lowers Lp(a) concentrations. Olpasiran reduces Lp(a) levels by 70–97% [72]. To investigate the efficacy and security of olpasiran in the clinical reduction of Lp(a), the OCEAN(a)-DOSE study is underway. The results have not yet been reported [112].

#### Inclisiran

Inclisiran is a siRNA inhibiting the synthesis of PCSK9. In ORION-1 (ClinicalTrials.gov, NCT02597127), inclisiran significantly reduced concentrations of LDL-C and PCSK9. Lp(a) concentrations also decreased in the group receiving inclisiran treatment [113]. A dramatic decrease in LDL-C concentrations was found in ORION-10 and 11 [114]. Because of the long biological half-life of inclisiran, twice-yearly dosing can result in a sustained lowering of LDL-C concentrations [115]. Clinical trials on inclisiran are ongoing.

### Somatic genome editing

**Fig. 3:**
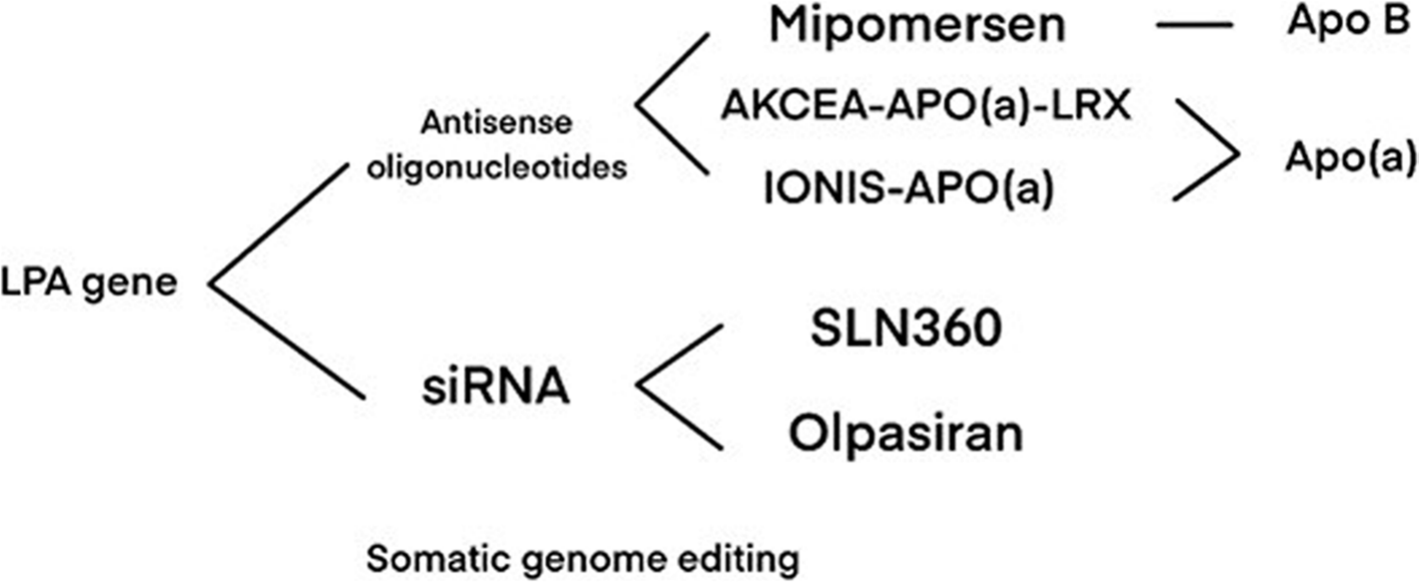
Summary of gene-based approaches under development

Alexandria M. Doerfler recently screened for CRISPR‒Cas9 genes and established a mouse model of LPA transgenic mice expressing the physiologic-related size apo(a). Adeno-associated virus (AAV) vector delivery of CRISPR‒Cas9 disrupts the LPA transgene in liver. Apo(a) was almost eliminated from circulation by AAV-CRISPR within one week. This trial suggests the viability of disrupting the LPA gene within the body using CRISPR‒Cas9 to reduce Lp(a) concentrations [116] Fig. 3.

## Discussion

Based on the studies discussed above in this review, it can be inferred that breast cancer survivors possibly have increased cardiovascular risk and disturbed lipid metabolism after CHT. In the studies by Saarto T [28] and Lu [30], Lp(a) concentrations were significantly increased in breast cancer survivors after CHT. However, in other subsequent related experiments, Lp(a) concentrations were mostly not notably altered. The experimental results are questionable because of the lack of a globally harmonized Lp(a) measurements. Because CHT regimens of the studies involved are not fully identical, the effects of different drugs on lipoproteins are different and may result in confounding results. It is difficult to separately assess the effect of a chemotherapeutic drug on lipids in the body because the paclitaxel drugs are used nearly exclusively in conjunction with anthracyclines for treatment. The influence of anthracyclines on lipid metabolism were long-lasting [39]. More precise experiments are needed in the future to explore the changes in Lp(a).

In Giskeødegård GF's study of longitudinal alterations in lipids after breast cancer therapies, they similarly revealed a progression toward atherogenic lipid signatures in all groups after therapy [37]. The cause of this abnormality in lipoprotein metabolism is not clear. On the one hand, CHT drugs may harm a number of normal tissue cells, leading to cellular oxidative stress as well as abnormal catabolism. On the other hand, ovarian failure due to CHT may also be the cause of dyslipidemia. Adriamycin (an anthracycline) decreased ABCA1 gene expression and apoA1 protein levels in hepatocytes. Since apoA1 and ABCA1 are essential for hepatic generation of HDL, this effect probably explains the relationship between adriamycin and decreased HDL levels [20]. Anthracyclines reduce HDL expression to inhibit its role in transporting cholesterol from vascular endothelium to the liver for recycling or elimination [117]. Paclitaxel promotes an increase in apoB expression and induces a decline in LDL receptor expression. These two actions may together lead to a disturbance in lipid metabolism and a greater prevalence of CVD [20]. Besides radiotherapy and chemotherapy, lifestyle changes in cancer survivors may lead to alterations in lipoprotein profiles through altered glutamate-glutamine metabolism [37].

Lipids are strongly associated with breast cancer progression and prognosis, but their effects are controversial [118]. In a study by Jung SM, breast cancer survivors with high LDL-C and low HDL-C concentrations suggested lower rates of cancer recurrence [119]. However, Dong S et al. followed 3499 women who were diagnosed with breast cancer. Their research showed that elevated TG concentrations at baseline and one year postoperatively raised the risk of recurrence, and increased HDL concentrations were associated with longer survival times. Both endocrine therapy and CHT can lead to elevated circulating lipid levels [120]. Mala Bahl also obtained similar results [121]. One speculation is that high cardiovascular mortality caused by dyslipidemia decreases breast cancer recurrence rates. The risk of dying from cardiovascular causes exceeds that of cancer death in elderly women with breast cancer [41]. Public still needs to pay attention to the risk of CVD. Endocrine therapy, obesity, and reduced exercise are also critical contributors to elevated incidence of CVD in breast cancer survivors.

The degree of reduction in Lp(a) concentrations that can influence CVD risk in clinical practice is still a question for exploration. A Mendelian randomization analysis evaluated the relationship between the incidence of coronary heart disease and Lp(a) concentrations in 48,333 subjects of European descent from 5 studies. This study found that lowering Lp(a) level by about 100 mg/dL has the potential to achieve a significantly decrease in the clinical risk of coronary heart disease, which is equivalent to the effect of decreasing LDL-C concentrations by 38.67 mg/dL with statins [9]. However, in a subsequent study by Claudia Lamina, the decline in Lp(a) concentrations was changed to 65.7 mg/dL in order to obtain the identical clinical outcome as the decrease in LDL-C of 38.67 mg/dL [122]. As number of meta-analyses and clinical studies to date to assess Lp(a) concentrations after CHT are minimal, more trials and literature are needed to support and explore this. The treatments currently available to decrease Lp(a) concentrations are suitable for patients with higher Lp(a) concentrations and an underlying disease.

A study by Cha J used a transgenic mouse that produces Lp(a) and found that Lp(a) probably has a function in the control of tumor growth and metastasis. The investigators believe that the probable mechanism of effect functions through competitive suppression of fibrin-induced extracellular matrix degradation by the Lp(a) component. This finding may reveal Lp(a) as an emerging and important target for tumor therapy [123]. Future studies on Lp(a) are promising to elucidate the specific mechanisms implicated by PSCK9i and method by which lowering Lp(a) affects CVD burden.

A clearer understanding of the connections among atherosclerosis, cancer treatment and lipid metabolism could provide the best lipid therapy for patients with cancer and reduce the burden of CVD. Monitoring of cardiotoxicity after breast cancer has not been resolved and there are no standard clinical guidelines to guide this [124]. Do Young Kim proposed The CHEMO-RADIAT score used to stratify cardiovascular risk in breast cancer survivors, which could help clinicians in their treatment decisions [125].

In addition to medication, positive lifestyle changes are the effective ways to prevent CVD and improve prognosis, such as psychological support [126], improving family support [127], proper nutrition [128] and exercises. Positive mental health has been determined to be prospectively associated with improved outcomes related to CVD [126].

## Conclusions and perspectives

Developments in neoadjuvant CHT contributed to an increase in breast cancer survivors. The determination of an appropriate follow-up for these patients is of increasing importance. An expanding body of evidence supports that various anticancer therapies may influence Lp(a) concentrations in breast cancer survivors. This review emphasizes the role of Lp(a) assessment and its implications in breast cancer care. Notably, this could be a possible risk factor for CVD in this population.

In addition, we provide an overview of the existing connection between cardiovascular prognosis and Lp(a) among patients with breast cancer receiving CHT. High Lp(a) levels may have adverse effects on their cardiovascular system. This review summarizes the treatments available to reduce Lp(a) concentrations in the clinical setting and the latest advances in the use of PSCK9 inhibitors. Also, we highlight the need to pay more attention to patients with breast cancer after CHT, who require safe and effective strategies for cardioprotection. Research on cardiac care for cancer survivors is still at its initial stage. Our findings need to be further investigated with more large-scale, prospective clinical trails, in patients with breast cancer receiving different classes of chemotherapeutic agents. Lipid-related effects on cardiovascular performance and well-being among cancer survivors have recently been an important research area. Governments and local communities should provide comprehensive health behavior counseling interventions for CVD prevention and management for many cancer survivors.

## Data Availability

Not applicable.

## Abbreviations

Lp(a): Lipoprotein(a)
Apo(a): Apolipoprotein a
ApoB: Apolipoprotein B-100
TG: Triglycerides
CAD: Coronary artery disease
CVD: Cardiovascular disease
CHT: Chemotherapy
LDL: Low-density lipoprotein
LDL-C: Low-density lipoprotein cholesterol
HDL: High-density lipoprotein
HDL-C: High-density lipoprotein cholesterol
LDLR: Low-density lipoprotein receptor
PCSK9: Proprotein convertase subtilisin/kexin-type 9
ASCVD: Atherosclerotic cardiovascular disease
